# Bone Lengthening Radius using Limb Reconstruction System - A Successful Treatment for Radius Shortening: A Case Report

**DOI:** 10.5704/MOJ.2411.013

**Published:** 2024-11

**Authors:** MR Muhammad-Zaidulkhair, RS Tan, IK Kamarul

**Affiliations:** 1 National Orthopaedic Centre of Excellence for Research and Learning (NOCERAL), Department of Orthopaedic Surgery, Universiti Malaya, Kuala Lumpur, Malaysia; 2 Department of Orthopaedics, Hospital Sultanah Bahiyah, Alor Setar, Malaysia

**Keywords:** radius shortening, bone lengthening, limb reconstruction surgery, monorail external fixator, paediatric

## Abstract

Fractures of the distal radius are the most common type of forearm fractures seen in children. The most serious outcome of physeal injuries is growth arrest, which can result in deformity and even significant differences in limb length. Therefore, we'd like to share our experience with treating a patient whose left radius stopped growing after she had a physeal injury in an accident. Case presentation: we encountered a 10-year-old girl, who was involved in a road traffic accident. She sustained closed fracture distal end left radius (Salter Harris 2). She sought medical assistance late, so osteoclasis, open reduction, and a k-wire on her left radius to fix the fracture, however it was complicated with growth arrest of left radius after the bone united. It was observed that her left radius was around 4cm shorter than her right. She had an osteotomy performed on her left radius and a LRS implanted. After six months post-surgery, there was no visible shortening of her left upper limb, and the radius had grown by around 4cm. There was no neurovascular impairment after left radius lengthening. After a year had passed after her operation, the patient said she had no complaints about her left upper limb. Despite the prevalence of the ilizarov method, the monorail external fixator, also known as LRS, is an option for bone lengthening of the radius. The LRS was utilised in our situation, and the results demonstrated its usefulness.

## Introduction

Fractures of the distal radius are the most common type of forearm fractures seen in children. The growth plate, also known as the physis, is a delicate portion of cartilage that can be seen in growing bones. The most serious and possibly life-threatening outcome of physeal injuries is growth arrest, which can result in deformity as well as a halt in longitudinal growth and even significant differences in limb length^1,2^.

Therefore, we'd like to share our experience with treating a patient whose left radius stopped growing after she had a physeal injury in an accident.

## Case Report

**Fig. 1:**
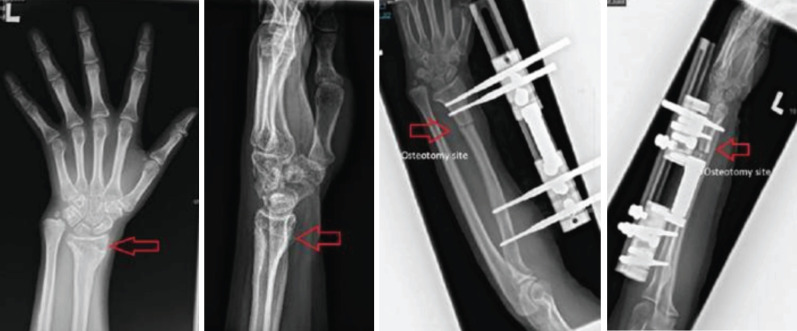
Radiograph on left – AP and lateral with left radius shortening radiograph on right -AP and lateral post osteotomy and LRS of left radius.

We encountered a 10-year-old girl, with no known medical illness, right hand dominant was involved in a road traffic accident. She sustained closed fracture distal end left radius (Salter Harris 2). She sought medical assistance late, so osteoclasis, open reduction, and a k-wire on her left radius to fix the fracture, however it was complicated with growth arrest of left radius after the bone unite (Fig. 1). It was observed that her left radius was around 4cm shorter than her right. She was limited in her ability to extend her wrist about 20° and performed palmar flexion about 30° due to the shortening. In addition, the radial deviation of the wrist deformity got worse over time. Patient had no limitation in terms of elbow flexion and extension. Consequently, she was planned for bone lengthening of left radius by a limb reconstruction system (LRS). She had an osteotomy performed on her left radius and a LRS implanted (Fig. 1).

**Fig. 2:**
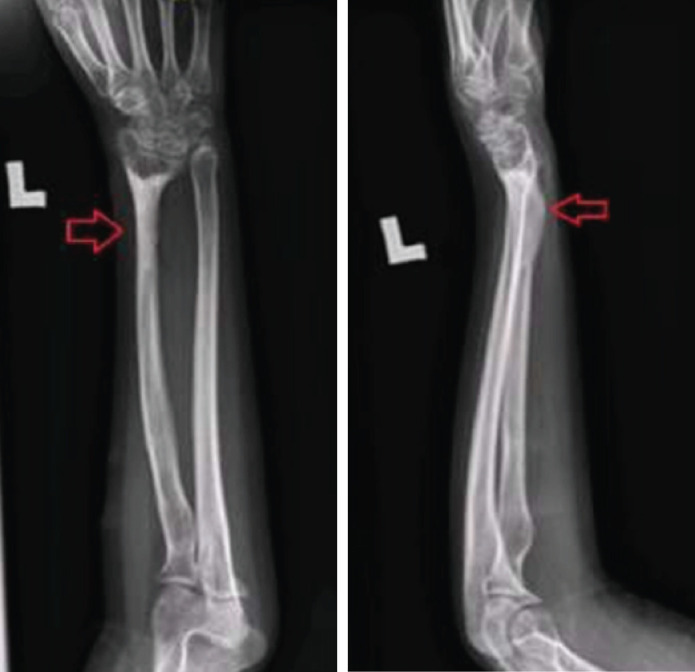
Radiograph after six months post-surgery right forearm after two months post-surgery.

**Fig. 3:**
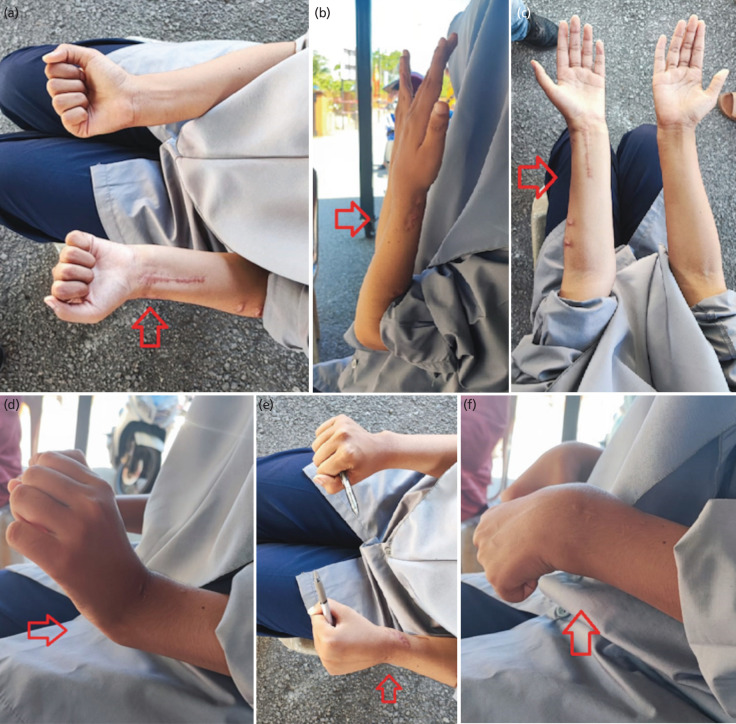
Range of motion of wrist and elbow post-surgery. (a) Forearm supination. (b) Elbow flexion. (c) Elbow extension. (d) Wrist extension. (e) Forearm pronation. (f) Wrist flexion.

By the tenth post-operative day, the incisions had closed, and the bone was started to lengthen at a rate of 1mm per day. Considering the necessity for a 4cm reduction in the left radius, the turn will take 40 days due to rate of lengthen is 1mm per day. LRS was maintained till radiological sign of union was obtained. During the process of elongation, the patient is capable of flexing her elbow and wrist in order to prevent stiffness in her joints. After 6 months post-surgery, there was no visible shortening of her left upper limb, and the radius had grown by around 4cm (Fig. 2). There was no neurological impairment after left radius lengthening, and her left upper limb had a detectable peripheral pulse. After a year had passed after her operation, the patient said she had no complaints about her left upper limb. The patient has achieved complete flexion and extension at the elbow and wrist, and both arms appear to be the same length, but there is some limitation to pronation. Similar handhold to the opposite side. The patient's DASH score was a -0.83 owing to the inability to analyse the patient's sexual activities, and she was allowed to resume her studies without any issues (Fig. 3).

## Discussion

Surgery may be necessary for forearm shortening, depending on the severity of the problem and how it affects functionality. The following reasons may necessitate surgery in our case: The functional limitations that our patient experienced impair hand function, which might affect daily activities and overall health. A common consequence of forearm shortening is wrist abnormalities such as radial deviation or restricted mobility. The alignment and functionality of the wrists and hands can be improved by surgically correcting these anomalies. Finally, the reason our patient needs surgery is to avoid complications. Sometimes, left untreated forearm shortening can lead to issues including nerve compression, deterioration of the joints, or persistent pain. Surgery may be necessary to avoid or minimise these issues and preserve long-term joint health. Furthermore, our patient is still quite young, and if the shortening is not treated, there's a chance she could get all the complications above.

We chose monorail fixator over ilizarov because it is less intrusive and causes less tissue injury. In addition, it is more convenient and pleasant because it is less bulky and lighter than the ilizarov. The monorail fixator is situated on the part of the radius that faces laterally. We used a total of four half-pins, with two being utilised in each clamp. This is because we positioned the monorail on a limb that did not contribute to the structure's weight, which provides an adequate amount of stability. In order to ensure that the lengthening occurs along the anatomical axis of the radius, we positioned the monorail parallel to the radius. The placement of monorail didn’t cross the wrist or elbow joints. This allowed the patient to do bone lengthening while maintaining range of motion in the wrist and elbow.

In conclusion despite the prevalence of the ilizarov method, the monorail external fixator, also known as LRS, is an option for bone lengthening of the radius.
